# Infants later diagnosed with autism have lower canonical babbling ratios in the first year of life

**DOI:** 10.1186/s13229-022-00503-8

**Published:** 2022-06-27

**Authors:** L. D. Yankowitz, V. Petrulla, S. Plate, B. Tunc, W. Guthrie, S. S. Meera, K. Tena, J. Pandey, M. R. Swanson, J. R. Pruett, M. Cola, A. Russell, N. Marrus, H. C. Hazlett, K. Botteron, J. N. Constantino, S. R. Dager, A. Estes, L. Zwaigenbaum, J. Piven, R. T. Schultz, J. Parish-Morris, The IBIS Network

**Affiliations:** 1 Center for Autism Research, Children’s Hospital of Philadelphia, Philadelphia, PA USA; 2 Department of Psychology, University of Pennsylvania, Philadelphia, PA USA; 3 Perelman School of Medicine, University of Pennsylvania, Philadelphia, PA USA; 4 National Institute of Mental Health and Neurosciences, Bangalore, India; 5 Department of Psychology, University of Texas at Dallas, Richardson, TX USA; 6 Department of Psychiatry, Washington University School of Medicine, St. Louis, MO USA; 7 University of North Carolina, Chapel Hill, NC USA; 8 University of Washington, Seattle, WA USA; 9 Department of Pediatrics, University of Alberta, Edmonton, Canada

## Abstract

**Background:**

Canonical babbling—producing syllables with a mature consonant, full vowel, and smooth transition—is an important developmental milestone that typically occurs in the first year of life. Some studies indicate delayed or reduced canonical babbling in infants at high familial likelihood for autism spectrum disorder (ASD) or who later receive an ASD diagnosis, but evidence is mixed. More refined characterization of babbling in the first year of life in infants with high likelihood for ASD is needed.

**Methods:**

Vocalizations produced at 6 and 12 months by infants (*n* = 267) taking part in a longitudinal study were coded for canonical and non-canonical syllables. Infants were categorized as low familial likelihood (LL), high familial likelihood diagnosed with ASD at 24 months (HL-ASD) or not diagnosed (HL-Neg). Language delay was assessed based on 24-month expressive and receptive language scores. Canonical babble ratio (CBR) was calculated by dividing the number of canonical syllables by the number of total syllables. Generalized linear (mixed) models were used to assess the relationship between group membership and CBR, controlling for site, sex, and maternal education. Logistic regression was used to assess whether canonical babbling ratios at 6 and 12 months predict 24-month diagnostic outcome.

**Results:**

No diagnostic group differences in CBR were detected at 6 months, but HL-ASD infants produced significantly lower CBR than both the HL-Neg and LL groups at 12 months. HL-Neg infants with language delay also showed reduced CBR at 12 months. Neither 6- nor 12-month CBR was significant predictors of 24-month diagnostic outcome (ASD versus no ASD) in logistic regression.

**Limitations:**

Small numbers of vocalizations produced by infants at 6 months may limit the reliability of CBR estimates. It is not known if results generalize to infants who are not at high familial likelihood, or infants from more diverse racial and socioeconomic backgrounds.

**Conclusions:**

Lower canonical babbling ratios are apparent by the end of the first year of life in ASD regardless of later language delay, but are also observed for infants with later language delay without ASD. Canonical babbling may lack specificity as an early marker when used on its own.

**Supplementary Information:**

The online version contains supplementary material available at 10.1186/s13229-022-00503-8.

## Introduction

Autism spectrum disorder (ASD) is a neurodevelopmental condition, which is diagnosed based on social communication differences and behavioral symptoms that generally emerge during the second and third years of life. However, some features of ASD may emerge earlier, within the first year of life. Canonical babbling—producing adult-like syllables with a consonant and vowel—is a speech–language skill that typically emerges in the first year, and disruption in the development of canonical babbling may be related to ASD [69]. Understanding the development of babbling in autism is important for three reasons. First, greater understanding of the development of language in autism is needed to understand the core social communication deficits that characterize the condition, as early speech–language differences can impact how an individual communicates. Second, some evidence examining group differences suggests that canonical babbling may have the potential to serve as an early behavioral marker of autism. Identifying such pre-diagnostic behavioral markers is an important goal, as reliable markers of likelihood could successfully prompt referral for early intervention, which is associated with better outcomes [21]. Third, identifying early developmental differences associated with ASD provides potential treatment targets, which is important because there are not yet evidence-based and established pre-symptomatic interventions for ASD [67], though one recent randomized clinical trial demonstrated efficacy [66]. Evidence indicates that toddlers who are diagnosed earlier have better outcomes at school age than those who receive intervention later [9], and treatment in the first year of life could leverage the plasticity of the infant brain to promote optimal development [62].

### Canonical babbling

Canonical babbling is an important, cross-cultural developmental milestone, which is achieved when infants regularly produce well-formed syllables including a consonant and vowel. Oller et al. [46], proposed the following model, which excludes both vegetative sounds (e.g., hiccups, coughs) and “fixed signals” (i.e., vocalizations that are functionally bound to a particular affective state, such as crying and laughing). The first 2 months of life are considered the phonation stage, in which infants produce quasivowels (partly resonant sounds produced with the vocal tract at rest) and glottals. Infants move into the primitive articulation stage by 2–3 months, wherein they produce vocalizations while moving the vocal tract (i.e., moving the lips, tongue, and pharynx to begin to articulate and alter vowel sounds), followed by the expansion stage in which infants produce full vowels (i.e., use their tongue, jaw, and lips to change resonance of the vocal tract) and marginal babbling (i.e., transitioning from a closed vocal tract to a full vowel, [46]). Finally, canonical babbling typically begins around 6 months and generally before 10 months, and is defined by the production of well-formed syllables. Well-formed syllables are further defined as a rapid formant transition between a consonant and a full vowel. This progression is thought to be relatively universal, and evidence supports that the typical age of canonical babbling onset across cultures occurs in the second half of the first year of life [11]. Infant babbling development is also thought to progress in a relatively standard order, with babies first producing canonical syllables which may be reduplicated (e.g., “baba”), followed by variegated babbling, or utterances which include two or more consonants (e.g., “bada”) [47]. Infants also acquire the ability to produce specific consonants in a relatively standard order across development [56], though language-specific differences in babbling emerge within the first year [5]. It has been suggested that babbling is a training ground for practicing multiple facets of communication: practicing the motor skills required to intentionally produce different sounds [26], practicing the most fundamental linguistic components of one’s language [50], and practicing communicative turn-taking “conversation” with others [1, 22].

Canonical babbling is thought to relate to subsequent language outcome. Delay in reaching this milestone (i.e., onset of babbling after 10 months) has been associated with reduced expressive vocabulary at 2.5 years [46]. Infants who have not reached the babbling milestone by 10 months are to be more likely than infants who do reach the milestone to have some kind of genetic, neurological, or developmental disability [45]. Reductions in babbling rates or complexity has been observed in late talkers [18, 57], and reduced canonical babbling has been associated with language-relevant developmental conditions, such as Fragile X and Rett syndrome [4, 28], with some individuals never reaching the canonical babbling stage [36]. In typically developing children, the age of onset of canonical babbling has been shown to predict the age of producing first words and expressive language at 18 months [39], although other studies have failed to detect a relationship between canonical babbling and word onset [17, 29]. A recent meta-analysis examining vocalizations and expressive language in ASD found a large effect for the relationship between consonant-centric measures and expressive language [37], and several reports document a longitudinal relationship between use of canonical syllables and expressive language in ASD [38, 68, 70].

Canonical babbling is typically measured as a proportion, with the numerator being the number of canonical syllables produced, and the denominator representing some total number, such as the number of utterances or syllables [43]. Such measures have been referred to as the canonical babbling ratio (CBR), canonical babbling proportion, and syllabic vocalization proportion (with vowel-only words, such as “I,” included in the numerator of the latter measure). Canonical babbling can also be measured as a milestone that has been achieved or not, by applying a cutoff to the canonical babbling ratio, such as 0.15 or greater [41], or by asking parents whether the behavior is demonstrated [45].

### Delays and reductions in canonical babbling associated with autism

Evidence indicates that autism is associated with babbling differences. Two studies examining toddlers with ASD found reduced babbling overall. In a sample of videotaped clinical interactions at 18 months, infants with ASD were significantly more likely than typically developing (TD) infants to produce no communicative vocalizations with consonants, though there was no difference when compared to infants with developmental delay [65]. However, this study was not able to disentangle consonant use from the likelihood of producing communicative vocalizations. In a recent study, vocalizations from 16- to 31-month-old children were collected via a 6-min tablet-based app [61]. Toddlers with ASD demonstrated a lower ratio of syllabic vocalizations (canonical vocalizations and vowel-only words such as “I”) compared to TD toddlers, but not developmentally delayed toddlers.

Infants at high familial likelihood(HL) for autism also have delays in canonical babbling compared to low- (LL) infants. One study of 5- to 14-month-old infants analyzed a combination of baby journals and monthly home recordings. Results revealed that a significantly greater proportion of HR infants were delayed in reaching the reduplicated babbling milestone (i.e., regularly repeating canonical syllables, such as “baba” or “gaga”) compared to LR infants [27]. Another study found a significant difference in the ratio of canonical syllables between HR and LR infants at 9 months as measured from the first 50 speech-like vocalizations of a parent–child interaction, though no differences were detected at 6 or 12 months [49]

Infants later diagnosed with autism have also been shown to have early delays in babbling, though results are somewhat mixed. Patten et al. [48] coded home videos of infants later diagnosed with ASD and TD aged 9–12 and 15–18 months old. The infants later diagnosed with ASD had lower canonical babbling ratios at both 9–12 months and 15–18 months. Additionally, they were less likely to have reached the canonical babbling stage at each age. In contrast to these findings, another study reported no differences in rates of reduplicated babbling or 2-syllable babbling at 0–6, 6–12, or 12–18 months in infants later diagnosed with ASD and TD infants [8]. In a study designed to assess developmental regression, home videos of first and second birthday parties were obtained for TD children, as well as children with ASD who did and did not have a parent-reported history of developmental regression [63]. These videos were coded for frequency of simple babbling, complex babbling, and words. At 12 months, there were no group differences in simple babbling, but group differences emerged in the frequency of complex babbling and word use combined into one count. Specifically, infants with ASD and regression showed the highest frequency of complex babbling and words at 12 months, followed by TD infants, and ASD infants without regression demonstrated the lowest complex babbling and word use. By 24 months, TD toddlers produced more complex babbling, single words, and 2-word phrases than both ASD groups, while simple babbling continued to show no group differences. This study highlights that developmental regression may be an aspect of heterogeneity that can cause differences in the development of babble in ASD, as 12-month-old infants with ASD and regression showed increased complex babbling, while those without regression showed reduced babbling at the same age. A combined ASD group (collapsing across regression and no regression) would likely have shown no differences.

Three studies directly compared babbling in autism versus developmental delays, with no differences reported [55, 61, 65]. This is not surprising, as differences in canonical babbling are associated with a range of developmental conditions. Combined with findings of differences between infants later diagnosed with ASD and TD infants, these studies suggest that canonical babbling may be a sensitive but not specific marker of autism.

Thus, while several studies suggest decreased canonical babbling ratios in infants later diagnosed with ASD relative to TD samples around 9 months, additional evidence is needed to establish the consistency of this pattern. Additionally, while the pattern of existing evidence suggests that differences in canonical babbling emerge at some point in the first year of life, longitudinal evidence is needed to assess whether developmental trajectories differ between groups.

### Predictive value of canonical babbling for ASD diagnosis

Importantly, there is some evidence that canonical babbling may be predictive of later autism diagnosis. In one study, discriminant function analysis was performed separately on data obtained at 6, 9, and 12 months to predict 24-month outcome in 14 HR infants later diagnosed with ASD (HR-ASD) and 11 HR infants not later diagnosed (HR-Neg) [49]. This model tested variables in which group differences were found: number of speech-like vocalizations, consonants, early/late/middle consonants, proportion of canonical syllables, and non-speech vocalizations. At 6 months, only the number of middle consonant types was predictive of later diagnosis, with a canonical correlation of 0.47 and 74% correctly classified. At 9 months, only the number of late consonant types was predictive (canonical correlation = 0.53, 77% accuracy). At 12 months, total number of different consonant types was predictive (canonical correlation = 0.43, 65% accuracy).

In a separate study, logistic regression was performed to test whether canonical babbling status (a ratio of 0.15 canonical babbling to total syllables) and speech-like volubility at 9–12 and 15–18 months predicted subsequent diagnosis between infants later diagnosed with ASD and TD infants [48]. A model including all four variables significantly predicted diagnosis with an overall accuracy of 75% (64% for TD, 82% for ASD), with only canonical babbling status at 9–12 months emerging as a significant independent predictor.

One recent study assessed the utility of 6-min samples of vocalizations collected via a tablet app to predict current diagnosis in 18–31-month-old toddlers [61]. The syllabic vocalization ratio showed high discriminability between ASD and TD toddlers, with an area under the curve (AUC) of 85.3. Discriminability between ASD and developmental delay was no better than chance (AUC = 57.4). Using a data-derived cut-point of 0.5, syllabic vocalization ratio achieved positive predictive value of 80% and negative predictive value of 73% for ASD versus no ASD. Infants with ratios below this cut-point (i.e., with less than 50% of vocalizations being syllabic) were 10 times more likely to have an ASD diagnosis. Together, these studies suggest that babbling may hold information that is useful for predicting later individual diagnostic outcomes.

### Present study

The goal of the present study was to examine canonical babbling ratios in a sample of infants at high and low familial likelihood for ASD. Speech-like vocalizations produced during semi-structured interactions with a clinician at 6 and 12 months of age were annotated for rates of canonical and non-canonical syllables. A strength of the present study is that it represents the largest HR-ASD sample to date (*n* = 44, compared to a range of 10–37 in the ASD/HR/HR-ASD samples in prior studies). Additionally, this sample is longitudinal. This allows for the analysis of change in babbling over time, as well as examination of the association between first-year CBR and language/diagnostic outcomes at age 2, which is important given prior findings of delayed babbling in non-ASD groups with language delay. We hypothesized that:

1. HR-ASD infants would have lower CBRs at each age compared to HR-Neg and LR infants.
2. HR-ASD infants would be less likely to be at the canonical babbling stage (as defined by CBR > 0.15) at each age compared to HR-Neg and LR infants.
3. HR-ASD infants would show slower rates of growth in canonical babbling between 6 and 12 months compared to HR-Neg and LR infants.
4. CBR would correlate with a standardized measure of expressive language at each age, and 12-month CBR would correlate with 24-month expressive language.
5. HR-Neg infants with language delay would have lower CBRs than LR infants, but similar to HR-ASD infants.
6. CBR at 6 and 12 months would significantly predict 24-month diagnostic outcome (ASD versus no ASD) in logistic regression.

## Methods

### Participants

Participants for this study were drawn from the larger Infant Brain Imaging Study (IBIS), a multisite longitudinal study funded by the National Institutes of Health Autism Centers of Excellence program. To be included in the high-likelihood (HR) group in IBIS, an infant was required to have an older sibling diagnosed with autism. Inclusion in the LR group required having at least one typically developing older sibling, and no first-degree relatives with autism or intellectual disability. General exclusion criteria for the IBIS study included genetic syndromes, medical or neurological conditions affecting growth, development, or cognition, significant sensory impairment, gestational age under 36 weeks, low birth weight, exposure to neurotoxins in utero, contraindication for MRI, predominant home language other than English, first-degree relative with psychosis, schizophrenia, or bipolar disorder, and twins. In the IBIS study, participants received social, cognitive/developmental, diagnostic evaluation and neuroimaging at 6, 12, and 24 months. Management of data collection, curation, and archiving was accomplished using the LORIS platform [13]. Inclusion criteria for the present study were: (1) an available audio–video recording at 6 or 12 months with at least one speech-like vocalization (see “Video sample”), (2) maternal education data, and (3) diagnostic outcome at 24 months. The sample reported here is a subset of all possible participants who met these inclusion criteria, which prioritized participants with both timepoints available, participants with neuroimaging data available, and those with videos which were available to the study team at the onset of data coding. See Table 1 for participant demographics.

**Table 1:** Demographic information for the full sample of infants with data at either 6 or 12 months (*N*’s provided separately at the bottom of the table)

	HR-ASD (*N* = 44)	HR-Neg (*N* = 141)	LR (*N* = 82)	*P*-value
*Sex*				
Female	6 (13.6%)	67 (47.5%)	28 (34.1%)	< 0.001
Male	38 (86.4%)	74 (52.5%)	54 (65.9%)	
*Maternal education*				
High School	2 (4.5%)	7 (5.0%)	3 (3.7%)	0.102
College	30 (68.2%)	90 (63.8%)	44 (53.7%)	
Graduate	12 (27.3%)	44 (31.2%)	35 (42.7%)	
*Race*				
Black/African-American	1 (2.3%)	5 (3.5%)	4 (4.9%)	0.46
More Than One Race	6 (13.6%)	16 (11.3%)	9 (11.0%)	
White	37 (84.1%)	117 (83.0%)	69 (84.1%)	
Asian	0 (0%)	3 (2.1%)	0 (0%)	
*Ethnicity*				
Hispanic	2 (4.5%)	9 (6.4%)	5 (6.1%)	0.416
Not Hispanic	42 (95.5%)	130 (92.2%)	77 (93.9%)	
Unknown/Not Reported	0 (0%)	2 (1.4%)	0 (0%)	
*24-Month MSEL expressive language T-score*				
Mean (SD)	36.4 (11.3)	49.1 (11.2)	52.9 (9.34)	< 0.001
Median [Min, Max]	36.0 [20.0, 58.0]	48.0 [25.0, 76.0]	54.0 [28.0, 73.0]	
Missing	1 (2.3%)	3 (2.1%)	0 (0%)	
*24-Month MSEL receptive language T-score*				
Mean (SD)	33.3 (16.2)	52.1 (10.2)	57.2 (8.11)	< 0.001
Median [Min, Max]	23.0 [20.0, 68.0]	52.0 [20.0, 74.0]	58.0 [30.0, 77.0]	
Missing	1 (2.3%)	3 (2.1%)	0 (0%)	
*24-Month language delay*				
No language delay	16 (36.4%)	121 (85.8%)	80 (97.6%)	< 0.001
Language delay	27 (61.4%)	17 (12.1%)	2 (2.4%)	
Unknown	1 (2.3%)	3 (2.1%)	0 (0%)	
*6-Month sample Ns*	*N* = 33	*N* = 105	*N* = 73	
*12-Month sample Ns*	*N* = 39	*N* = 129	*N* = 71	

Language delay was determined by 24-month Mullen expressive and receptive language scores. *P* values indicate results of an ANOVA for continues demographic variables and a Chi-square test for categorical variables

### Behavioral assessment

Two standardized language measures were available. At 6, 12, and 24 months, infants were administered the Mullen Scales of Early Learning (MSEL, [42]). This standardized developmental assessment measures five domains (visual reception, fine motor, gross motor, receptive language, and expressive language) in children through age 68 months. Although not a dedicated speech–language measure per se, the MSEL Expressive Language scale assesses a broad range of expressive language components, including aspects of speech production, vocabulary, gesture use, and grammar. At 12 and 24 months of age, parents completed the Macarthur-Bates Communicative Development Inventories (M-CDI), Words and Gestures form [12]. While the Words and Gestures form is not standardized for 24-month infants, the raw number of words produced serves as a consistent estimate of vocabulary across development, and was used in analyses.

Diagnostic evaluation was conducted at 24 months, and included administration of the Autism Diagnostic Observation Schedule (ADOS-2, [32]) and Autism Diagnostic Interview-Revised (ADI-R, [33]). ASD diagnoses were based on best clinical estimate, with expert clinicians using all available information to apply DSM-IV-TR criteria. Infants meeting criteria for autism or pervasive developmental disorder-not otherwise specified (PDD-NOS) were considered to have ASD in keeping with DSM-5 criteria. The HR group was divided into infants who received an ASD diagnosis (HR-ASD) and those who were negative for ASD at 24 months (HR-Neg). Infants in the LR group were required to be negative for ASD at 24 months [24].

Because infants at high familial likelihood for ASD are also at higher risk for language disorders, follow-up analyses were conducted splitting the HR-Neg and HR-ASD groups into those with and without language delay. The language delay groups were defined as infants with a 24-month MSEL Expressive Language or Receptive Language T-score of less than 35 [59, 60]. The LR group was not split, as only two children met these criteria for language delay in the LR group, and these children were retained in the LR group. Thus, there were five groups: HR-ASD with language delay (HR-ASD-LD), HR-ASD without language delay (HR-ASD-No), HR-Neg with language delay (HR-Neg-LD), HR-Neg without language delay (HR-Neg-No), and LR. Full demographic data for these groups are in Additional file 1: Table S1.

### Video sample

Infants completed the Autism Observation Scale for Infants (AOSI, [6]) at 6 and 12 months. The AOSI is a brief (10–15 min), developmentally appropriate interaction, designed to elicit behaviors relevant to ASD, such as social babbling, response to name, imitation, and transitions. At 12 months, infants also completed the Communication and Symbolic Behavior Scales (CSBS, [64]). The CSBS is a 20–30 min play session designed to assess social communication, expressive/receptive language, and symbolic functioning. All interactions were video and audio recorded. At 12 months, the CSBS was selected for annotation if it was completed and available. The CSBS was selected because it is longer than the AOSI and thus provides more data, and also to facilitate longitudinal comparisons with vocalizations produced at 24 months during the CSBS as part of a larger coding effort [51]. If a CSBS was not available (due to recording failure or the missing/incomplete administration), the AOSI was used. To ensure results were not driven by differences in behavioral sample, sensitivity analyses were conducted excluding data derived from the 12-month AOSI.

### Coding

Annotations were completed using a reliable coding system. First, each infant vocalization was segmented (i.e., start and end times marked) by a trained rater using ELAN annotation software. A second trained rater reviewed the entire file for accuracy and corrected any errors. “All vocalizations produced by the infant were segmented and annotated, regardless of whether the vocalization appeared to have communicative intent (e.g., by combining vocalizations with gaze or gesture directed toward another person, or not).” After segmentation, two trained raters independently annotated each vocalization as speech-like, non-speech, or vegetative. Speech-like segments were defined as sounds with recognizable phonemes (e.g., babbling). Non-speech sounds were defined as vocalizations characterized by resonance and vocal quality not typical of speech, often without recognizable phonemes [49, 52, 53, 55]. Vegetative sounds do not have linguistic or semantic intent and are produced naturally (e.g., burping or coughing) and were not further analyzed in the present study. Discrepancies were resolved through consensus (i.e., raters discussed differences and came to agreement). Rater training included a presentation on the coding pipeline, a requirement that raters establish greater than 80% agreement on a set of gold-standard training reliability files, and a requirement to attend periodic reliability meetings. This pipeline is described further in [51].

Only segments labeled as speech-like were further coded for canonical babbling. That is, vocalizations initially coded as non-speech or vegetative were not coded for canonical babbling. Canonical babbling ratio is typically defined as:

$${\text{CBR }} = \frac{{{\mathrm{Canonical\,syllables}}}}{{{\mathrm{Canonical\,syllables}} + {\mathrm{non-canonical\,syllables}}}}$$

Canonical syllables were defined as a syllable meeting the following criteria: “a fully-resonant nucleus, at least one supraglottally articulated (e.g., tongue, lip, or jaw) consonant-like element (i.e., a margin), and a timely formant transition between the nucleus and the margin,” following Oller [44, p. 114]. Non-canonical syllables included syllables without a margin (i.e., consisting of vowels only), syllables with only glottal or fricatives as margins, marginal babbles (i.e., syllables meeting two of the three criteria described for canonical babbles), and syllables consisting only of supraglottally generated sounds. Utterances labeled as canonical babbling were further coded for the type of babble, with options of reduplicated babbling, variegated babbling, or neither. Reduplicated babbling was defined as repeated perceived syllables, as in “mama.” Variegated babbling was defined as utterances with two or more canonical syllables in which the perceived syllables were different, as in “mami” or “mana.” Utterances not meeting either of these criteria (e.g., a single canonical syllable) were labeled as “neither.”

Raters were trained by reading a chapter on babbling [7], reviewing a training manual, listening to labeled clips, completing an online training (http://www.babyvoc.org/IVICT.html), and coding several training files with feedback. Raters established a minimum reliability by establishing an ICC of at least 0.80 with a gold standard (a set of three training test files labeled by the first author) for both number of syllables and number of canonical syllables on before they began coding, and participated in ongoing reliability meetings.

Raters were instructed to rate all segments from a timepoint at once. Raters first watched and listened to the first 10 segments from a timepoint to familiarize themselves with the infant and adults’ voices, and then rated each segment individually. For each segment, the rater determined: (1) the number of syllables, (2) the number of canonical syllables, and (3) the type of babbling. Raters had the ability to replay the audio-visual or audio-only of the segment as many times as they desired. A rating of “uncodable” was assigned only if the infant could not be heard, or if the rater felt that the segment did not contain a speech-like vocalization. This occurred primarily if the vocalization contained only a gasp, which was categorized as speech-like in level 1 coding but does not qualify as a syllable under most definitions of canonical babbling ratio. Raters were blinded to diagnostic outcome group. Each file was rated by two coders independently. A third rater resolved discrepancies by selecting between the two original ratings. In the case that the third rater felt neither of the original ratings was accurate, the first author reviewed the segment to make a final determination. Due to the COVID-19 pandemic and resulting loss of ability for students to complete coding, the first author resolved discrepancies for 210 timepoints. Among these files, the first author selected a response other than one of the original codes in the case of only 25 segments.

Reliability was measured in two ways. First, a subset of 26 files spanning 6, 12, and 24 months was entirely double-coded by separate sets of raters to determine the reliability of the segmentation and initial annotation pipeline. The reliability for the number of speech-like vocalizations was ICC(*A*,1) = 0.89. Additionally, a set of 30 files (5 per group at each age) was randomly selected and entirely double-coded by two separate sets of raters (e.g., two distinct pairs of independent raters, and a different discrepancy-resolver). The CBR was calculated from each set of ratings and compared, with ICC(*A*,1) = 0.84 overall (ICC(*A*,1) = 0.66 for 6-month-olds and ICC(*A*,1) = 0.81 for 12-month-olds). This overall level of reliability is in line with other reports on CBR in infants (e.g., [30, 31, 48]). As noted later, the moderate reliability observed in 6-month-olds may be related to the small number of vocalizations produced by many infants during the recording sessions at this age.

### Analysis

Some children produced a small number of speech-like vocalizations at a given timepoint, which may impact CBR estimates [41]. To mitigate this risk, we applied 3-sigma exclusion based on CBR for the given timepoint. This resulted in the exclusion of 3 HR-Neg and 4 LR infants at 6 months, and no infants at 12 months.

All analyses included sex, site, and maternal education as covariates, with diagnostic group (HR-ASD, HR-Neg, LR) as the term of interest with HR-ASD as the reference group. To examine canonical babbling ratio, generalized linear models were used with a binomial distribution to account for the binary nature of the data (“canonical” or “non-canonical”). To examine the effects of language delay, similar analyses were conducted using the diagnostic language groups (HR-ASD-LD, HR-ASD-No, HR-Neg-LD, HR-Neg-No, LR), with Tukey-corrected pairwise comparisons between groups to examine all pairwise effects. To assess canonical babbling onset, a cutoff of 0.15 CBR was used, which has been used in prior studies [4], 30, 31, 48. Likelihood of achieving canonical babbling onset was assessed by calculating the odds ratio. The relationship between CBR and standardized language measures was also assessed by generalized linear models with a binomial distribution. Rates of reduplicated and variegated babbling were analyzed with zero-inflated generalized linear models with a Poisson distribution to account for the count nature of the data and high number of infants who produced no examples of reduplicated or variegated babble. Recording duration was used as an offset which accounts for differences in recording times by creating a covariate with an assumed coefficient of 1, allowing for an analysis of the rate of reduplicated or variegated babble per minute of recording time [25].

To assess the predictive value of canonical babbling for 24-month diagnostic outcomes, a logistic regression was fit on the full sample using only 6-month and 12-month CBR as predictors, controlling for site, sex, and maternal education. This analysis focused on the full sample rather than accuracy scores obtained from cross-validated model training because the present study is focused on a single behavior. It is unlikely that any single behavior would have high predictive accuracy for subsequent autism diagnosis. The goal of this analysis was therefore to obtain estimates of the predictive value of the individual variables.

## Results

### No significant group differences in CBR at 6 months

There was no significant difference in CBR between groups at 6 months (HR-ASD *M* = 0.03, *SD* = 0.04; HR-Neg *M* = 0.02, *SD* = 0.04; LR *M* = 0.02, *SD* = 0.03). Sex was the only significant covariate (site and maternal education were non-significant). Male sex was associated with higher CBR (*p* < 0.01). There was no difference in the likelihood of reaching the canonical babbling milestone by group at 6 months (HR-Neg *OR* = 0.734, *p* = 0.74; LR *OR* = 0.000000007, *p* = 0.995). Of note, 51.5% of infants produced no canonical syllables during the recording, with a range of 0–14 canonical syllables per infant. Even fewer utterances consisting of reduplicated or variegated babbles were observed, with ranges of 0–2 and 0–1, respectively. Given the extremely low number of observations of these behaviors, group comparisons in rates of reduplicated or variegated babbling were not justified (Table 2).

**Table 2:** Means, standard deviations, and ranges of types of syllables and utterances

	6 Months			12 Months		
	HR-ASD	HR-Neg	LR	HR-ASD	HR-Neg	LR
Recording duration	13.14 (2.95), 8.08–18.80	13.06 (3.16), 7.07–23.25	13.35 (3.72), 7.87–28.54	20.33 (6.18), 7.79–38.31	20.23 (4.99), 8.90–37.49	19.27 (3.99), 9.42–32.78
Number of speech-like vocalizations	25.78 (19.70), 3–81	20.87 (16.01), 2–91	17.84 (13.82), 2–83	31.25 (26.78), 6–185	34.78 (17.82), 2–177	32.52 (14.41), 9–127
Number of syllables	49.91 (38.41), 7–178	36.60 (32.57), 2–201	34.34 (33.69), 2–181	54.73 (40.40), 8–584	64.97 (33.74), 4–309	59.07 (29.95), 10–353
Number of canonical syllables	1.65 (2.29), 0–12	1.09 (1.87), 0–11	1.24 (2.37), 0–14	9.28 (10.58), 0–130	16.01 (15.82), 0–175	14.17 (14.84), 0–203
Canonical babbling ratio	0.03 (0.04), 0–0.17	0.02 (0.04), 0–0.20	0.02 (0.03), 0–0.12	0.16 (0.12), 0–0.42	0.22 (0.15), 0–0.63	0.22 (0.16), 0–0.58
Number of reduplicated utterances	0.10 (0.31), 0–2	0.07 (0.24), 0–1	0.12 (0.36), 0–2	0.82 (1.07), 0–9	1.63 (2.09), 0–24	1.62 (2.27), 0–20
Number of variegated utterances	0.14 (0.31), 0–1	0.03 (0.14), 0–1	0.05 (0.20), 0–1	0.96 (1.68), 0–21	1.87 (2.81), 0–35	1.46 (2.53), 0–38

All means and standard deviations (except recording duration and canonical babbling ratio) are presented as counts per 10 min, to account for differing recording times. Raw ranges are presented to provide an accurate depiction of the size of the samples of vocalizations

### HR-ASD infants have lower CBR at 12 months

**Fig. 1:**
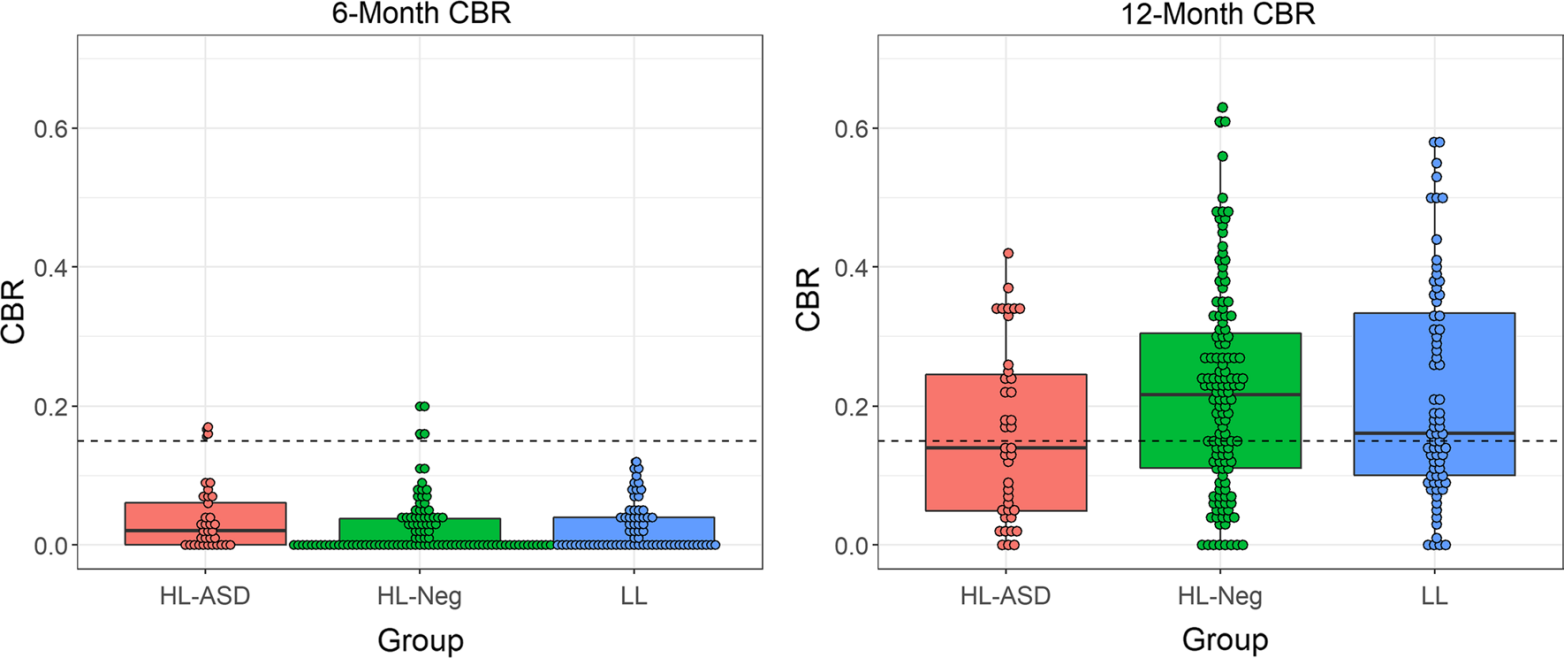
Canonical babbling ratios (CBRs) at 6 and 12 months. The dashed line indicates the commonly used threshold of 0.15, above which infants are considered to have reached the canonical babbling milestone. Dark lines show the median for each group

There were significant differences in CBR between groups at 12 months. HR-ASD produced a lower CBR compared to both HR-Neg (HR-ASD *M* = 0.16, *SD* = 0.12; HR-Neg *M* = 0.22, *SD* = 0.15; Estimate = 0.32, *p* < 0.0001) and LR (*M* = 0.22, *SD* = 0.16, Estimate = 0.33, *p* < 0.001). All covariates (site, sex, and maternal education) were significant predictors of CBR. Higher CBR was associated with female sex, higher maternal education, and evaluation at the St. Louis and Seattle sites (relative to Philadelphia). Notably, as shown in Fig. 1, all groups showed high variability, with CBR ranging from 0 to 0.63. As a follow-up to examine whether significant differences in this ratio were driven by the numerator (canonical syllables) or the denominator (non-canonical syllables + canonical syllables), two GLMs were fit examining the effect of diagnosis on each count (canonical or non-canonical syllables), controlling for site, sex, maternal education, and recording time using a Poisson distribution. There were significant differences in the rate of producing canonical syllables between groups, with HR-Neg infants (Estimate = 0.31, *p* < 0.0001) and LR infants (Estimate = 0.29, *p* < 0.0001) producing more canonical syllables than HR-ASD infants. In contrast, there were no significant differences in the rate of production of non-canonical syllables between HR-ASD and other groups (*p*s > 0.05).

In contrast to the significantly lower CBR observed in the HR-ASD group, there was no difference in the likelihood of reaching the canonical babbling milestone by group at 12 months (HR-Neg *OR* = 1.68, *p* = 0.18; LR *OR* = 1.23, *p* = 0.62). Of note, a smaller-than-expected number of infants in the LR group had reached the canonical babbling milestone (55%) using the traditional definition of CBR > 0.15. To explore the possibility that a lower cutoff is more appropriate for our relatively short recordings, the 10th percentile of CBR for the LR group was also used as a cutoff (CBR > 0.05), and there remained no difference in likelihood of reaching the milestone by group.

Because of the high number of individuals who produced no reduplicated or variegated babbles, zero-inflated regression was used to examine differences in these types of babble. There were significant differences in rates of reduplicated babble. The HR-Neg group was significantly less likely than the HR-ASD group to produce zero reduplicated babbles (*OR* = 0.42, *p* = 0.049). Zero reduplicated babbles were produced by 51% of the HR-ASD group (*n* = 20), 28% of the HR-Neg group (*n* = 36), and 31% of the LR group (*n* = 22). Among infants who produced reduplicated babbles, the LR group produced 1.36 times more than the HR-ASD group (*p* = 0.038). There were no significant differences in rates of producing variegated babbles. The number of infants producing zero variegated babbles by group was: HR-ASD *n* = 20 (51%), HR-Neg *n* = 39 (30%), LR *n* = 26 (37%).

To ensure that results at 12 months were not driven by the subset of infants for whom data were derived from the AOSI rather than the CSBS (*n* = 19), analyses of CBR, babbling milestone attainment, and reduplicated/variegated babbling were re-run with these individuals excluded, with a nearly identical pattern of results and negligible changes to effect estimates (see Supplemental Materials). The only change to significance observed was in the analysis of reduplicated babble, in which the findings just described were no longer significant (zero-inflation HR-ASD relative to HR-Neg, *p* = 0.074, HR-ASD relative to LR count, *p* = 0.086).

### Growth in babbling over time

**Fig. 2:**
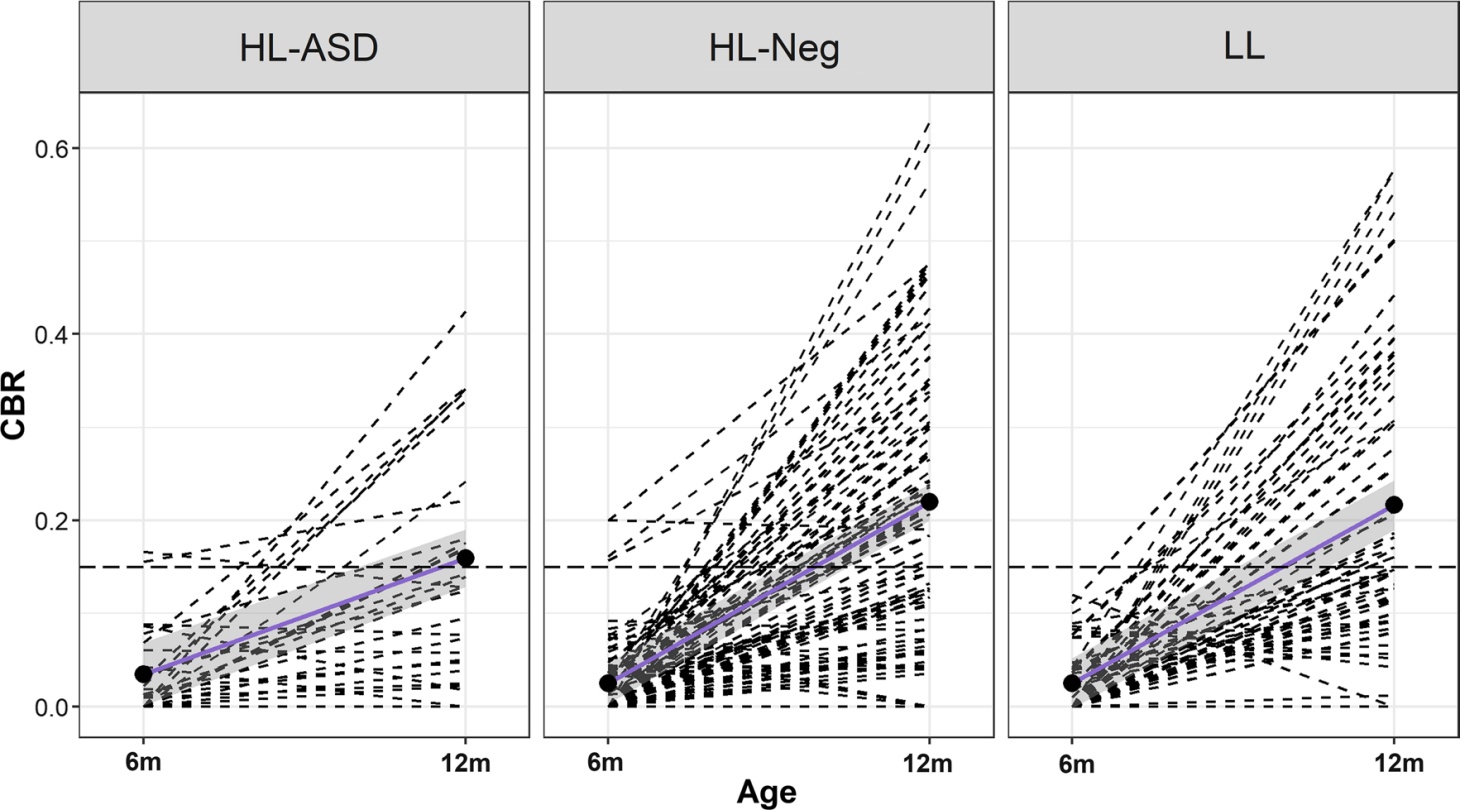
Longitudinal change in canonical babbling ratio. Dashed lines represent individual trajectories, and the long-dashed line indicates the commonly used canonical babbling milestone cut-point of 0.15 for reference. HR-ASD infants show significantly reduced growth in CBR between 6 and 12 months relative to HR-Neg and LR groups

There were significant group-by-age interactions, indicating differing rates of growth in CBR between 6 and 12 months by group. Specifically, compared to the HR-ASD group, faster rates of growth in CBR were observed in the HR-Neg (Estimate: 0.11, *p* < 0.001) and LR (Estimate = 0.086, *p* < 0.01) groups. Visual inspection of Fig. 2 reveals significant individual variability in CBR growth, with a few individuals across groups showing declines over time.

### Relationship with standardized language measures across groups

**Fig. 3:**
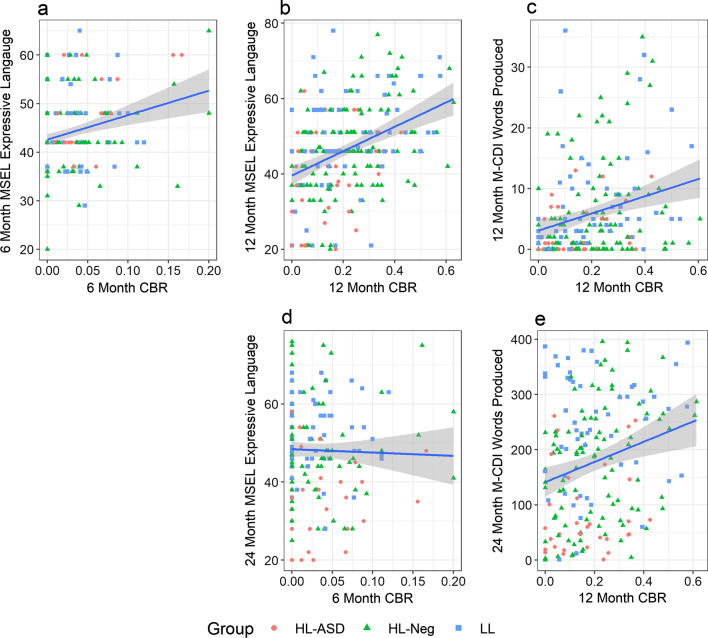
CBR was significantly associated with standardized language measures, controlling for sex, site, and maternal education. **a** CBR at 6 months was predictive of MSEL Expressive Language at 6 months. CBR at 12 months was predictive of: **b** MSEL Expressive Language at 12 months, **c** M-CDI words produced number at 12 months, **d** MSEL Expressive Language measured at 24 months, and **e** M-CDI words produced number at 24 months

The relationship between CBR and standardized language measures was assessed across all infants for whom scores were available (see Additional file 1: Table S2 for scores and *N*s). At both 6 and 12 months, CBR was significantly associated with concurrently measured MSEL Expressive Language (6 months *η*_*p*_^2^ = 0.08, *p* < 0.001; 12 months *η*_*p*_^2^ = 0.13, *p* < 0.0001, Fig. 3). At 12 months, extreme outliers in M-CDI words produced number were apparent upon visual inspection. Two-sigma outlier removal was applied (see Additional file 1: Figure S1). CBR measured at 12 months was significantly associated with the number of words produced as measured by the M-CDI at 12 months (*η*_*p*_^2^ = 0.06, *p* < 0.001). Notably, 12-month CBR was also a significantly associated with 24-month MSEL Expressive Language (*η*_*p*_^2^ = 0.03, *p* < 0.01) and with 24-month M-CDI words produced (*η*_*p*_^2^ = 0.04, *p* < 0.01). Exploratory analyses were conducted to examine the relationship between 6-month CBR and later language measures. CBR at 6 months was not significantly associated with the MSEL EL at 12 months (*η*_*p*_^2^ = 0.0038, *p* = 0.80) or 24 months (*η*_*p*_^2^ = 0.00037, *p* = 0.80), nor with the M-CDI words produced at 24 months (*η*_*p*_^2^ = 0.0013, *p* = 0.66).

### Language delay

**Fig. 4:**
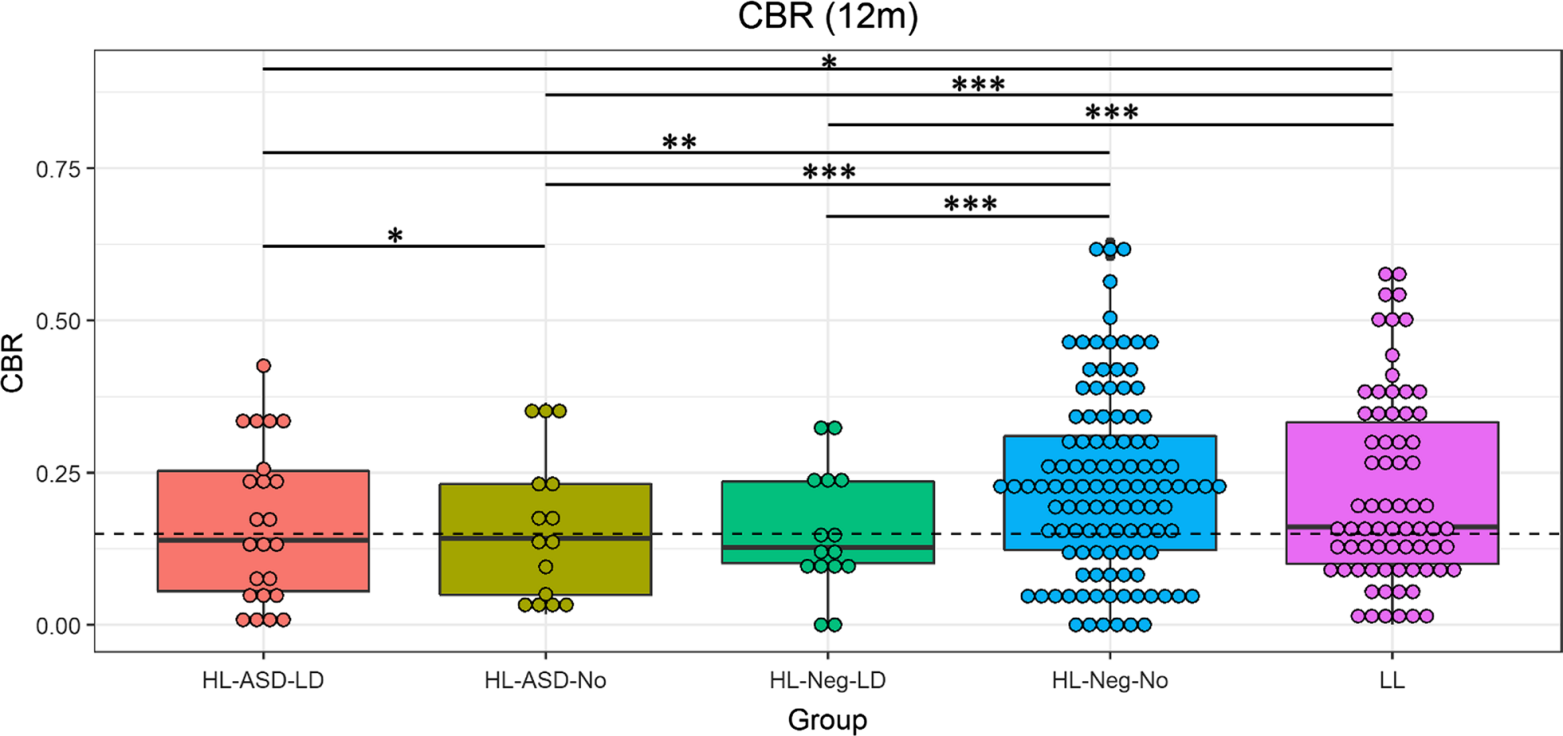
Canonical babbling ratios (CBRs) at 12 months by likelihood, diagnostic, and language delay grouping. High-likelihood infants with language delay but not autism (HR-Neg-LD) were not distinguished from high-likelihood infants with autism but not language delay (HR-ASD-No). High-likelihood infants with neither autism nor language delay (HR-Neg-No) and low-likelihood (LR) infants had higher CBR than HR-ASD-No infants. Asterisks indicate significance in Tukey-corrected pairwise comparisons from a GLM controlling for site, sex, and maternal education, *p < 0.05, **p < 0.01, ***p < 0.001

The HR groups were divided into infants with language delay (HR-Neg-LD *N* = 17 and HR-ASD-LD *N* = 27) and without language delay (HR-Neg-No *N* = 121 and HR-ASD-No *N* = 16), see Fig. 4 and Table 3. A generalized linear mixed model was fit controlling for site, sex, and maternal education, with Tukey-corrected pairwise comparisons between groups. (Full tables of pairwise comparisons can be found in Additional file 1: Table S3.) At 6 months, two significant differences emerged. HR-Neg-LD produced higher CBR than HR-ASD-No (Estimate = 0.91, *p* < 0.05). That is, contrary to expectations, the high-likelihood infants who went on to have language delays but not ASD demonstrated higher CBR than those who did not later have language delays or ASD. Additionally, HR-Neg-LD produced higher CBR than HR-Neg-No (Estimate = 0.60, *p* < 0.05).

**Table 3:** Means and standard deviations of CBR at 6 and 12 months by diagnostic and language group

	6-Month CBR	12-Month CBR
HR-ASD-LD	0.04 (0.05)	0.17 (0.13)
HR-ASD-No	0.02 (0.02)	0.16 (0.12)
HR-Neg-LD	0.04 (0.04)	0.15 (0.10)
HR-Neg-No	0.02 (0.04)	0.23 (0.15)
LR	0.02 (0.03)	0.22 (0.16)

At 12 months, there were many significant differences in CBR between groups. The LR group produced significantly higher CBR than all other groups *except* HR-Neg-No (Estimate = -0.03, *p* = 0.86). That is, all infants with either ASD or LD produced lower CBR than LR infants, while HR infants *without* language delay or ASD produced typical CBR. Notably, there was not a significant difference in CBR produced by HR-Neg-LD and HR-ASD-No (Estimate = 0.04, *p* = 0.99) or between HR-Neg-LD and HR-ASD-LD (Estimate = -0.20, *p* = 0.12). That is, HR infants who did not go on to receive an ASD diagnosis but *did* demonstrate language delays produced similar CBR as infants later diagnosed with ASD (with or without language delay). HR-ASD infants produced reduced CBR compared to LR, regardless of whether they also had delayed language.

### Prediction

Logistic regression was used to assess the predictive value of 6- and 12-month CBR for ASD outcome at 24 months, controlling for site, sex, and maternal education in all infants with data at both timepoints. CBR values were multiplied by 100 to produce interpretable odds ratios. The odds ratios provided can be interpreted as representing the odds of receiving an ASD diagnosis associated with a one percentage point change in the proportion of canonical babbling (e.g., a change of CBR = 0.14 to CBR = 0.15). In this model, neither 6-month CBR (OR = 1.02, *p* = 0.67) nor 12-month CBR (OR = 1.03, *p* = 0.07) was a significant predictor of ASD outcome. Results were similar when the logistic regression for ASD outcome was conducted within the HR group only (i.e., excluding the LR infants) using the same covariates (6-month CBR OR = 1.01, *p* = 0.82, 12-month CBR OR = 1.03,* p* = 0.08). To explore the relative contributions of CBR and standardized language measures to ASD prediction, MSEL Expressive Language scores at 6 and 12 months were added to the full-sample logistic regression. In this model, neither 6- nor 12-month CBR was a significant predictor of ASD outcome, but 12-month MSEL Expressive Language was (OR = 1.07, *p* = 0.04).

## Discussion

In one of the largest studies to date examining canonical babbling in infants at high familial likelihood for ASD, we found that reduced CBR was associated with ASD by age 1, but this effect is nonspecific, as CBR was also associated with language delay. At 12 months of age, HR-ASD infants showed reduced CBR relative to both HR-Neg and LR infants, partly supporting hypothesis 1. This finding is consistent with several prior reports of delayed canonical babbling in ASD [27, 48, 49, 63, 65]. This reduction in CBR was driven by the HR-ASD group producing lower rates of canonical syllables (the numerator of CBR) than HR-Neg and LR groups, in the context of producing a similar rate of non-canonical syllables. That is, when 12-month-old infants later diagnosed with ASD produce speech-like vocalizations, these vocalizations are less likely to be mature canonical syllables. Additionally, we find support for hypothesis 3, with the HR-ASD group showing slower growth in CBR between 6 and 12 months relative to the LR and HR-Neg groups.

When the high-likelihood groups were sub-divided into those infants who do and do not have language delay, HR-Neg infants with language delay produced similar CBR to HR-ASD infants with or without language delay, as predicted by hypothesis 5. This finding is consistent with prior studies, which have found differences in babbling between ASD and TD infants, but not ASD and developmentally delayed infants [55, 61, 65]. As discussed, delayed canonical babbling is associated with language delay [46]. Notably, HR-ASD infants *without* language delay produced lower CBR than HR-Neg infants without language delay, LR infants, and HR-ASD infants with language delay. This indicates that lower CBR in ASD is not merely a result of higher rates of language delay in this population, but is associated with autism itself. Thus, although CBR is not a specific marker of autism, it indicates increased likelihood of both autism and language delay, and early intervention is recommended in both cases.

Although canonical babbling is not likely to be specific enough to predict later autism diagnosis on its own (demonstrated by the failure to support hypothesis 6 through logistic regression), it holds the potential to be a useful marker in combination with other early signs. Differences in canonical babbling emerge notably early in development. In the present study, they were observed by 12 months and in prior work have been observed by 9 months [48, 49]. Additionally, efforts are underway to automate detection of canonical babbling [54], which would make this behavior relatively easy and inexpensive to measure. Canonical babbling may be most useful as an early predictor in conjunction with a more specific autism marker, for example, whether infants direct their vocalizations toward others.

Canonical babbling ratio was related to standardized language measures, in support of hypothesis 4. Notably, 12-month CBR was significantly associated with 24-month standardized language scores. Babbling is thought to be an important precursor to the acquisition of words, as canonical syllables are the building blocks of words. Delays in canonical babbling have previously been associated with subsequent language delays [39], although such relationships are not always detected [17, 29]. It should be noted that 12 months are later than the window during which canonical babbling typically emerges, and that delay in babbling would more ideally be measured at a time in between the timepoints used in this study (e.g., 7–10 months). Indeed, many infants are speaking their first words by 12 months of age and are past the canonical babbling stage, although in our coding scheme canonical syllables produced as part of words are still counted as canonical, and infants were therefore not penalized for this linguistic maturity. Despite the timing of the measurement, this finding confirms that CBR as measured from a brief laboratory-based interaction is associated with subsequent language development.

Differences in babbling were not detected at 6 months, in contrast to the prediction in hypothesis 1. Two prior studies examining this age range have failed to find differences between HR and LR infants [49] or between TD infants and infants later diagnosed with ASD [8]. To our knowledge, no prior studies have found significant differences in canonical babble associated with ASD at this age. Children typically achieve the canonical babbling milestone between 6 and 10 months [44]. Therefore, it is expected that very few infants will demonstrate canonical babbling when measured at 6 months. It may be that 6 months are simply too early to detect differences in canonical babbling between infants who go on to receive an ASD diagnosis compared to those who do not, because the skill has not yet emerged. Another possibility is that the null group differences were due to the small number of speech-like utterances produced by infants in this study. While all infants produced at least one speech-like vocalization (which is necessary to calculate CBR), some produced a relatively small number. For example, 17.6% of infants produced fewer than 10 speech-like vocalizations. Therefore, it cannot be ruled out that differences could be detected with a larger speech sample. This may require longer recording durations to capture a sufficient number of speech-like vocalizations, which could be achieved through home recordings such as the day-long recordings obtained using the LENA system (e.g., [59, 60]. Such a study could more conclusively determine whether babbling differences emerge by this early age. From a practical perspective, the failure of any study to date to detect canonical babbling differences at this age is at the very least a strong indication that canonical babbling as assessed from a relatively brief recording at 6 months is unlikely to serve as a useful clinical predictor.

No group differences in the likelihood of reaching the canonical babbling milestone at 6 or 12 months were detected, failing to support hypothesis 2. This contrasts with prior findings of delays in reaching the canonical babbling milestone in HR infants relative to LR infants [27] and in infants later diagnosed with ASD [48], as well as currently unpublished findings from the current sample using day-long home recordings obtained at 9 months (Meera et al., in prep). The traditional definition of achieving the milestone—CBR of at least 0.15—has been useful in prior studies (e.g., [4, 30, 31, 48]). For example, 10-month-olds with CBR of less than 0.15 had smaller vocabularies later in development [46]. Though grounded in data, this precise cut-point is somewhat arbitrary, and different definitions may be appropriate for different samples and different measurement methods and contexts. Nonetheless, we did not detect differences in the likelihood of achieving the milestone even with a much lower cutoff. Statistically, dichotomizing a continuous variable reduces power [10]. This suggests that (1) efforts to employ babbling measures for early detection at a given age would be better served by using CBR than a binarized babbling onset, and (2) it is important to have roughly similar recording lengths and situations, as the CBR is sensitive to these variables.

High variability in CBR was observed across groups at 12 months and in growth trajectories between 6 and 12 months. Notably, some LR infants who demonstrated very low CBR at 12 months nonetheless had high scores on the MSEL Expressive Language scale or M-CDI words produced at 24 months. This variability may be due to methodological limitations associated with using a single laboratory-based assessment of CBR (e.g., it is unlikely that the small number of individuals showing a decrease in CBR between 6 and 12 months demonstrated a true regression in CBR). High variability may also relate to the fact that some infants show a transient delay in language development and then catch up (late talkers), while others demonstrate consistent delays [35]. Additionally, the patterns observed in this study may reflect underlying variability in language development across infants in the general population, with previous reports suggesting a “fan effect” of increasing variability in language ability beginning around 12 months of age (as measured by words produced, [19]. Finally, variability may be associated with several measured and unmeasured additional factors. For example, at 12 months in the current study, higher CBR was associated with: female sex (language is often reported to be more advanced in females than males (e.g., [3, 16, 20, 23]), though this is confounded in by the sex imbalance in the HR-ASD group); higher maternal education (which has been associated with more advanced language development (e.g., [15, 34])) and site, which may relate to minor administration differences or underlying demographic differences between sites.

Mechanistically, three functions of babble have been proposed: motor practice [26], linguistic component practice [50], and communicative turn-taking practice [1, 22]. Any or all of these three forms of practice may increase an infant’s later language skills. The third form of practice—conversational turn-taking—may be of particular relevance for infants at elevated likelihood of developing ASD, as social communication differences, including difficulties with back-and-forth conversation, are a core diagnostic feature of ASD [2]. Furthermore, speech exposure and opportunities to practice conversational turn-taking are driven by the home language environment, which could differ between LR and HR groups due to shared genetic differences between parents and children, parental behavior changes due to having an older child on the spectrum, or differences evoked by the infants themselves (e.g., temperament). In day-long home recordings from a sample that partly overlaps with the current sample, LR infants were found to have more growth in conversational turn-taking from 9 to 15 months of age than HR infants [58]. Future research should directly investigate the relationship between home language exposure, conversational turn-taking, and canonical babbling.

## Limitations

As discussed, the relatively short recording times and resulting low numbers of speech-like vocalizations in this sample may impact the reliability of our estimates of canonical babbling ratio, particularly at 6 months of age. While reliability of the behavioral coding in this study was assessed to be good, only one recording was used to measure canonical babbling for each infant at each timepoint, and so the test–retest reliability of this measure is unknown. It is possible that the selection of available videos biased the sample in some unknown way (e.g., because children with particularly strong behavioral difficulties were unable to complete the assessments). Additionally, the sample is not representative of the general global population, as it is over 80% White, over 90% non-Hispanic, and over 90% of mothers in the sample had at least some college education. Furthermore, the infant sibling design limits the generalizability of findings to the general population. In the general population, language delay is much more prevalent than ASD [40], whereas this sample was specifically enriched for ASD. Additionally, there are potential underlying differences between HR-ASD infants and infants later diagnosed with ASD without an older sibling with ASD [14].

## Conclusion

This study suggests that canonical babbling is an early-emerging but nonspecific behavioral marker of autism, which may hold clinical utility for early diagnosis if combined with other early bio-behavioral markers. Lower canonical babbling ratios were observed in HR-ASD infants as compared to HR-Neg and LR infants by 12 months, though this difference was not yet evident at 6 months and occurs in the context of significant individual variability in CBR. A subset of HR-Neg infants with delayed language at 24 months also showed lower canonical babbling ratios at 12 months, similar to the HR-ASD group. This study indicates the need for future work investigating the sensitivity and specificity of canonical babbling in conjunction with other early markers of ASD in predicting diagnostic outcomes.

## Supplementary Information

**Additional file 1: Supplementary Material**. Supplementary figure, tables, and text.

## Data Availability

The dataset generated during and analyzed during the current study is not publicly available due the parameters of parent/participant consent, but is available from the corresponding author on reasonable request.

## Abbreviations

ASD: Autism Spectrum Disorder
CBR: Canonical Babbling Ratio
HL-ASD: High Likelihood, with Autism Spectrum Disorder diagnosis
HL-ASD-LD: High Likelihood, with Autism Spectrum Disorder diagnosis, with Language Delay
HL-ASD-No: High Likelihood, with Autism Spectrum Disorder diagnosis, no Language Delay
HL-Neg: High Likelihood, with no Autism Spectrum Disorder diagnosis
HL-Neg-LD: High Likelihood, with no Autism Spectrum Disorder diagnosis, with Language Delay
HL-Neg-No: High Likelihood, with no Autism Spectrum Disorder diagnosis, no Language Delay
LL: Low Likelihood
M-CDI: Macarthur-Bates Communicative Development Inventories
MSEL: Mullen Scales of Early Learning
