# Supplementary material for: Infants later diagnosed with autism have lower canonical babbling ratios in the first year of life
Source: Mol Autism. 2022 Jun 27;13:28. doi: 10.1186/s13229-022-00503-8 (PMC9235227; doi:10.1186/s13229-022-00503-8)
Supplement: Supplementary file 1 — Additional file 1: Supplementary Material. Supplementary figure, tables, and text. [file 13229_2022_503_MOESM1_ESM.docx]

Table S1. Demographic information for infants with data at 6 or 12 months, separated by diagnostic group and presence of language delay at 34 months.

**Sensitivity analysis of canonical babbling at 12 months excluding infants who received the AOSI at 12 months**

To ensure that results at 12 months were not due to differences related to rating the vocalization sample from the AOSI rather than the CSBS, 12-month analyses were re-run using only infants who received the CSBS at 12 months (HL-ASD *n* = 35, HL-Neg *n* = 121, LL *n* = 65). There were significant differences in CBR between groups at 12 months. HL-ASD produced a lower CBR compared to both HL-Neg (HL-ASD *M* = 0.15, *SD* = 0.12; HL-Neg *M* = 0.22, *SD* = 0.15; Estimate = 0.31, *p* < 0.0001) and LL (*M* = 0.23, *SD* = 0.16, Estimate = 0.36, *p* < 0.001). All covariates (site, sex, and maternal education) were significant predictors of CBR.

In contrast to the significantly lower CBR observed in the HL-ASD group, there was no difference in the likelihood of reaching the canonical babbling milestone by group at 12 months (HL-Neg *OR* = 1.87, *p* = 0.13; LL *OR* = 1.52, *p* = 0.34).

Because of the high number of individuals who produced no reduplicated or variegated babbles, zero-inflated regression was used to examine differences in these types of babble. There were not significant differences in rates of reduplicated babble, whereas significant differences were detected when using the full (AOSI and CSBS) sample. The HL-Neg group was no longer significantly less likely than the HL-ASD group to produce zero reduplicated babbles (*OR* = 0.44, *p* = 0.074). Zero reduplicated babbles were produced by 49% of the HL-ASD group (*n* = 17), 28% of the HL-Neg group (*n* = 34), and 28% of the LL group (*n* = 18). Among infants who produced reduplicated babbles, the LL group produced 1.29 times more than the HL-ASD group, but this effect was no longer significant (*p* = 0.086). There were no significant differences in rates of producing variegated babbles. The number of infants producing zero variegated babbles by group were: HL-ASD *n* = 19 (54%), HL-Neg *n* = 38 (31%), LL *n* = 22 (34%).

Table S2. Standardized language scores associated with babbling samples at 6- and 12-months.

| **6-Month CBR** | **HL-ASD (N=33)** | **HL-Neg (N=105)** | **LL (N=73)** | **P-value** |
| --- | --- | --- | --- | --- |
| **6-Month MSEL Expressive Language T-score** |  |  |  |  |
| Mean (SD) | 45.4 (7.33) | 43.2 (7.29) | 44.6 (7.38) | 0.222 |
| Median [Min, Max] | 42.0 [36.0, 60.0] | 42.0 [20.0, 65.0] | 42.0 [29.0, 65.0] |  |
| **12-Month CBR** | **HL-ASD (N=39)** | **HL-Neg (N=129)** | **LL (N=71)** | **P-value** |
| **12-Month MSEL Expressive Language T-score** |  |  |  |  |
| Mean (SD) | 39.4 (12.1) | 46.7 (11.5) | 49.6 (12.3) | <0.001 |
| Median [Min, Max] | 42.0 [20.0, 62.0] | 46.0 [20.0, 77.0] | 51.0 [21.0, 78.0] |  |
| Missing | 0 (0%) | 1 (0.8%) | 0 (0%) |  |
| **24-Month MSEL Expressive Language T-score** |  |  |  |  |
| Mean (SD) | 37.2 (11.0) | 48.3 (10.3) | 53.1 (9.59) | <0.001 |
| Median [Min, Max] | 37.0 [20.0, 58.0] | 47.5 [28.0, 75.0] | 54.0 [28.0, 73.0] |  |
| Missing | 1 (2.6%) | 3 (2.3%) | 0 (0%) |  |
| **12-Month M-CDI Words Produced Number** |  |  |  |  |
| Mean (SD) | 2.97 (3.92) | 8.11 (18.8) | 7.67 (8.24) | 0.178 |
| Median [Min, Max] | 1.00 [0, 13.0] | 3.00 [0, 172] | 5.00 [0, 36.0] |  |
| Missing | 3 (7.7%) | 21 (16.3%) | 14 (19.7%) |  |
| **24-Month M-CDI Words Produced Number** |  |  |  |  |
| Mean (SD) | 83.2 (78.0) | 175 (106) | 242 (102) | <0.001 |
| Median [Min, Max] | 48.0 [1.00, 261] | 191 [0, 396] | 260 [1.00, 394] |  |
| Missing | 6 (15.4%) | 30 (23.3%) | 17 (23.9%) |  |


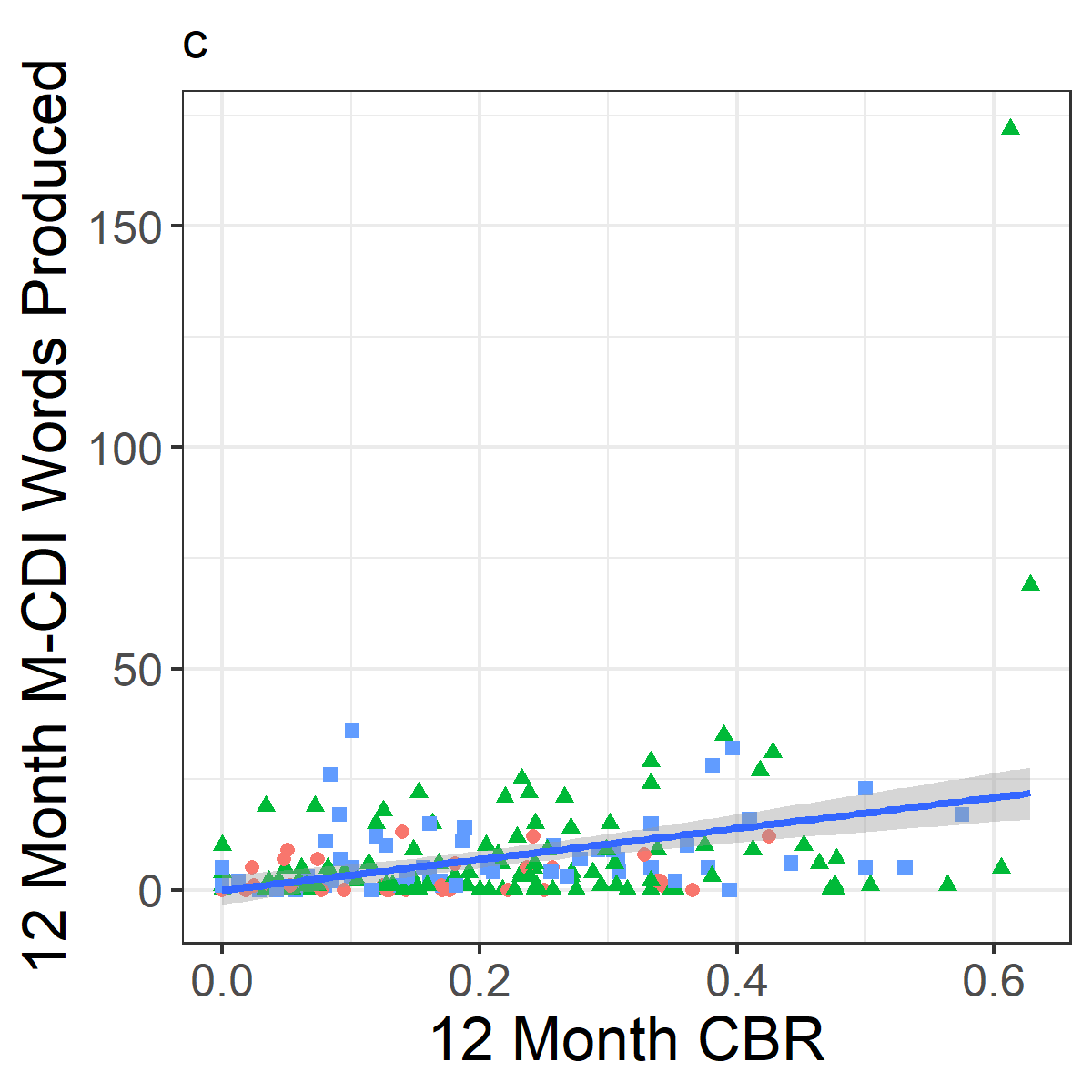


Figure S1. Two outliers are apparent in the M-CDI Words Produced number, and were excluded from analyses. When these outliers are included, 12-month CBR remains significant associated with 12-month M-CDI Words produced, controlling for site, sex, and maternal education (η_p_^2^ = 0.11, *p* < 0.0001)

| **6 Months** | **Estimate** | **Std. Error** | **z value** | **Pr(>\|z\|)** |  |
| --- | --- | --- | --- | --- | --- |
| HL-ASD-LD - HL-ASD-No == 0 | 0.7062 | 0.2757 | 2.562 | 0.0729 | . |
| HL-Neg-No - HL-ASD-No == 0 | 0.3043 | 0.2576 | 1.181 | 0.752 |  |
| HL-Neg-LD - HL-ASD-No == 0 | 0.909 | 0.2977 | 3.054 | 0.0176 | * |
| LL - HL-ASD-No == 0 | 0.4608 | 0.257 | 1.793 | 0.3639 |  |
| HL-Neg-No - HL-ASD-LD == 0 | -0.4019 | 0.1798 | -2.235 | 0.1581 |  |
| HL-Neg-LD - HL-ASD-LD == 0 | 0.2029 | 0.2265 | 0.896 | 0.8932 |  |
| LL - HL-ASD-LD == 0 | -0.2453 | 0.177 | -1.386 | 0.6238 |  |
| HL-Neg-LD - HL-Neg-No == 0 | 0.6047 | 0.2049 | 2.952 | 0.0243 | * |
| LL - HL-Neg-No == 0 | 0.1565 | 0.142 | 1.102 | 0.7966 |  |
| LL - HL-Neg-LD == 0 | -0.4482 | 0.206 | -2.176 | 0.1792 |  |
| **12 Months** | **Estimate** | **Std. Error** | **z value** | **Pr(>\|z\|)** |  |
| HL-ASD-LD - HL-ASD-No == 0 | 0.23353 | 0.07988 | 2.923 | 0.0255 | * |
| HL-Neg-No - HL-ASD-No == 0 | 0.45775 | 0.06095 | 7.51 | < 0.001 | *** |
| HL-Neg-LD - HL-ASD-No == 0 | 0.03731 | 0.08569 | 0.435 | 0.99167 |  |
| LL - HL-ASD-No == 0 | 0.42635 | 0.06343 | 6.721 | < 0.001 | *** |
| HL-Neg-No - HL-ASD-LD == 0 | 0.22423 | 0.05881 | 3.813 | 0.00119 | ** |
| HL-Neg-LD - HL-ASD-LD == 0 | -0.19622 | 0.08378 | -2.342 | 0.11976 |  |
| LL - HL-ASD-LD == 0 | 0.19282 | 0.06139 | 3.141 | 0.01309 | * |
| HL-Neg-LD - HL-Neg-No == 0 | -0.42045 | 0.06729 | -6.248 | < 0.001 | *** |
| LL - HL-Neg-No == 0 | -0.03141 | 0.03263 | -0.962 | 0.86044 |  |
| LL - HL-Neg-LD == 0 | 0.38904 | 0.06945 | 5.602 | < 0.001 | *** |

Table S3. Tukey-corrected pairwise comparisons of CBR between diagnostic and language groups.

Note. Significance codes: 0 ‘***’ 0.001 ‘**’ 0.01 ‘*’ 0.05 ‘.’ 0.1 ‘ ’ 1
